# Defective Autophagy, Mitochondrial Clearance and Lipophagy in Niemann-Pick Type B Lymphocytes

**DOI:** 10.1371/journal.pone.0165780

**Published:** 2016-10-31

**Authors:** Barbara Canonico, Erica Cesarini, Sara Salucci, Francesca Luchetti, Elisabetta Falcieri, Gianna Di Sario, Fulvio Palma, Stefano Papa

**Affiliations:** 1 Department of Biomolecular Sciences, University of Urbino Carlo Bo, Urbino, Italy; 2 IGM, CNR, Rizzoli Orthopaedic Institute, Bologna, Italy; Department of Biomolecular Sciences, University of Urbino Carlo Bo, Urbino, Italy; University of Alabama at Birmingham, UNITED STATES

## Abstract

Niemann-Pick disease type A (NP-A) and type B (NP-B) are lysosomal storage diseases (LSDs) caused by sphingomyelin accumulation in lysosomes relying on reduced or absent acid sphingomyelinase. A considerable body of evidence suggests that lysosomal storage in many LSD impairs autophagy, resulting in the accumulation of poly-ubiquitinated proteins and dysfunctional mitochondria, ultimately leading to cell death. Here we test this hypothesis in a cellular model of Niemann-Pick disease type B, in which autophagy has never been studied. The basal autophagic pathway was first examined in order to evaluate its functionality using several autophagy-modulating substances such as rapamycin and nocodazole. We found that human NP-B B lymphocytes display considerable alteration in their autophagic vacuole accumulation and mitochondrial fragmentation, as well as mitophagy induction (for damaged mitochondria clearance). Furthermore, lipid traceability of intra and extra-cellular environments shows lipid accumulation in NP-B B lymphocytes and also reveals their peculiar trafficking/management, culminating in lipid microparticle extrusion (by lysosomal exocytosis mechanisms) or lipophagy. All of these features point to the presence of a deep autophagy/mitophagy alteration revealing autophagic stress and defective mitochondrial clearance. Hence, rapamycin might be used to regulate autophagy/mitophagy (at least in part) and to contribute to the clearance of lysosomal aberrant lipid storage.

## Introduction

Niemann-Pick disease (NPD) consists of a group of genetic disorders in which the common feature is a varying degree of lipid storage in certain tissues of the body. In particular, Niemann-Pick types A/B are caused by a recessive mutation in the SMPD1 gene encoding acid sphingomyelinase (ASMase), resulting in sphingomyelin accumulation in lysosomes. Niemann-Pick type A (NP-A) is a severe neurodegenerative disorder of infancy, which is usually fatal by 3 years of age, whereas Niemann-Pick B (NP-B) patients have minimal or no neurologic involvement and often survive into adulthood [1]. This disorder falls into the category of lysosomal storage diseases (LSDs). LSDs comprise nearly 60 different inherited disorders, caused by the inability of the lysosomal system to degrade specific metabolites, resulting in abnormal storage/accumulation within the lysosome. As a consequence, many tissues and organs are affected, with early onset neurodegeneration within the central nervous system predominating [2].

Autophagy is an intracellular lysosomal degradation and recycling process characterized by the formation of a double membrane-bound vesicle called the autophagosome, which plays a role in the bioenergetic management of starvation [3]. Autophagy is central to the process of cellular quality control, removing waste or excess proteins and organelles. Excessive organelle damage and degradation, related impairments of autophagolysosomal maturation, and differences in co-activated pathways and cellular context may determine whether activation of autophagy plays a pro-survival or pro-death role [4].

Recently, there has been increasing attention focused on the autophagic pathway in lysosomal storage diseases (LSDs). Such interest is based on the hypothesis that accumulation of undegraded substrates in lysosomes, due to the deficiency of specific lysosomal enzymes, may impair the autophagic process. An alteration in autophagy has been shown in many LSDs, including Niemann-Pick disease type A [5], Niemann-Pick type C (NP-C), Mucopolysaccharidosis type IIIA (MPS-IIIA), Multiple Sulphatase Deficiency (MSD) and Danon disease [6]. In particular, a marked accumulation of autophagosomes and ubiquitinated proteins occurs in the brain of Niemann-Pick type A mice and in fibroblasts from NP-A patients [5], and an amassing of elongated and unclosed autophagic membranes has been found in NP-A fibroblasts [7]. In LSDs, multiple mechanisms, resulting in dysregulated or imbalanced induction, maturation, or degradation, converge to create the pathologic condition of “autophagic stress” [8], characterized by sustained increases in autophagic vacuoles (AVs).

Damaged mitochondria might be autophagocytosed selectively in a process called mitophagy [9,10]: this process is thought to play a role in maintaining a healthy mitochondrial population via removal of dysfunctional/depolarized mitochondria. There is a growing consensus that alterations in mitochondrial function and dynamics, as well as in the mitophagic pathway, are involved in a number of neurodegenerative diseases such as Huntington’s and familial forms of Parkinson’s disease [11]. Furthermore, accumulation of fragmented or altered mitochondria has been observed in cells from several LSDs and in normal cells treated with inhibitors of autophagy or lysosomal function [12,13].

To date, the mitophagy pathway has never been studied in Niemann-Pick types A/B, *in vivo* or *in vitro* models. In the present study, we examined the progression of autophagy/mitophagy in a cell model represented by EBV-transformed ASMase^-/-^ B lymphocytes, in which we investigated alterations of autophagic/mitophagic features contributing to this pathologic state. Lymphocytes were chosen as our *in vitro* model because of their straightforwardness as a non-adherent cell line (contrary to fibroblasts) and because they afford the possibility of repeating specific tests on blood cells from patients in the future.

In addition, we analyzed the effects of the autophagy modulating substances Rapamycin (RM), Nocodazole (NZ) and Wortmannin (WM) on autophagic vacuole formation. During this exogenous autophagic modulation we performed an accurate screening by means of flow cytometry (FC), confocal microscopy (CM) and transmission electron microscopy (TEM) of the following range of cellular responses: cell viability/cell death; mitochondrial integrity and function; lysosomal, endosomal and autophagosomal compartments; mitochondrial autophagy (mitophagy) induction; intracytoplasmic lipid content; microvesicles and lipid particles in extracellular environment.

The results of these evaluations point to the possible use of the above-mentioned drugs to modulate the autophagy/mitophagy imbalance (at least in part) and to limit the basal autophagic stress of this peculiar cellular model, maintaining good cell viability and a regular intracytoplasmic arrangement.

## Results

### Flow Cytometric evaluation of cell death parameters

We used two different B lymphocyte cell lines: one from a NP-B patient (referred to as tNP—transformed Niemann-Pick) and the second from a healthy donor (referred to as tWT—transformed wild-type). Both lines were treated by specific inducer/inhibitors (see Material and Methods section for references): rapamycin (inducing autophagy by mTOR complex inhibition), wortmannin (a PI3K inhibitor) and nocodazole, (provoking microtubule depolymerization). To induce further autophagy, we also grew cells in starvation.

**Fig 1:**
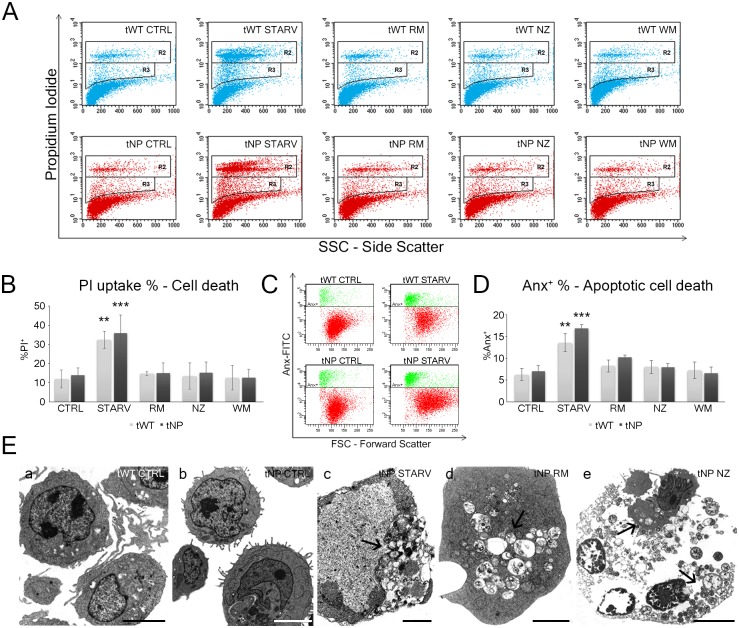
Evaluation of cell death by Propidium Iodide (PI), Annexin (Anx) and transmission electron microscopy. (**A**) Dot plots PI *vs* SSC from Niemann-Pick (tNP) and wild-type (tWT) B lymphocytes for control (CTRL), starved (STARV), rapamycin (RM), nocodazole (NZ) and wortmannin (WM) treated cells. Rectangular regions show events with low PI uptake (PI^+^ in R3) and high PI uptake (PI^++^ in R2), pointing to apoptosis and necrosis, respectively. (**B**) The statistical histogram shows the total PI uptake percentage (events in R2 and R3) in non-pathological and pathological lymphocytes. Each value is expressed as a percentage ± SD (Results from n ≥ 3 independent experiments); **P < 0.01 and ***P < 0.001 *vs* respective control. (**C**) Dot plots Annexin-FITC *vs* FSC from Niemann-Pick (tNP) and wild-type (tWT) B lymphocytes from control (CTRL) and starved (STARV) cells. Rectangular regions (populations in green) show Anx^+^ early apoptotic events. (**D**) The statistical histogram shows Annexin (Anx) positivity percentage in non-pathological and pathological lymphocytes. Each value is expressed as a percentage ± SD (Results from n ≥ 3 independent experiments); **P < 0.01 and ***P < 0.001 *vs* respective control. (**E**) TEM images of tWT (**a**) and tNP (**b**-**e**) cells from different experimental conditions: in control condition (**a**, **b**), after nutrient deprivation (**c**), rapamycin (**d**) and nocodazole (**e**) administration. In **c**, **d** and **e** autophagic cell death in vacuolated cells is recognizable (arrows: autophagic vacuoles, AVs). Bars: 5 μm for a, b, e; 2 μm for c, d.

Autophagy has often been described as a pro-survival pathway, as it allows the recycling of damaged or undamaged cellular elements and can also directly regulate positively or negatively several forms of cell death, including apoptosis, necrosis, necroptosis, and pyroptosis [14]. Accordingly, we first evaluated cell death in untreated and inducer/inhibitor-treated cells from both cell lines, by flow cytometry using supravital Propidium Iodide (PI) staining (Fig 1A). The statistical histogram based on the cytometric data (Fig 1B) showed that there were no significant differences between normal and pathological cells in terms of PI uptake, revealing the lack of death features after inducer/inhibitor administration. Only starvation affected cell viability, with a PI^+^ cell percentage of about 35% in both cell lines (Fig 1A and 1B). Supravital PI uptake revealed both necrotic and apoptotic pathways in starved cells. Annexin analyses (Fig 1C and 1D) confirmed the supravital PI data, showing no significant differences between tWT and tNP Anx positivity and the greatest apoptotic response in starved cells. Moreover, ultrastructural analyses confirmed the cytometric observations: in particular, apoptotic chromatin condensation as well as necrotic features were revealed. After all treatments, a diffuse cytoplasmic vacuolization, pointing to the presence of autophagic vacuoles, can be observed. Indeed, several autophagic-like vacuoles were observed in these cells, evidence of lysosomal substrate accumulation, a typical feature of lysosomal storage diseases (Fig 1E). In addition, TEM revealed autophagic cell death features in tNP cells, particularly in those that underwent starvation treatment.

### Analysis of mitochondrial integrity and functions highlights specific dysfunctional features

As mitochondria balanced survival-death stimuli, we performed an analysis of cellular mitochondria content, focusing on functional and dysfunctional mitochondria by means of flow cytometry and TEM. These methods effectively reveal possible mitochondrial alteration and/or fragmentation. It should be noted, however, that mitochondrial morphology is dynamic, and mitochondrial fragmentation precedes apoptosis in several systems [15,16].

Hence, although we did not find any specific evidence of apoptosis or necrosis after inducer/inhibitor administration, we carefully analysed the mitochondrial network and status. In fact, since LSD cells are more sensitive to apoptosis, the possibility remains that mitochondrial fragmentation in our cell model reflects pre-apoptotic changes.

**Fig 2:**
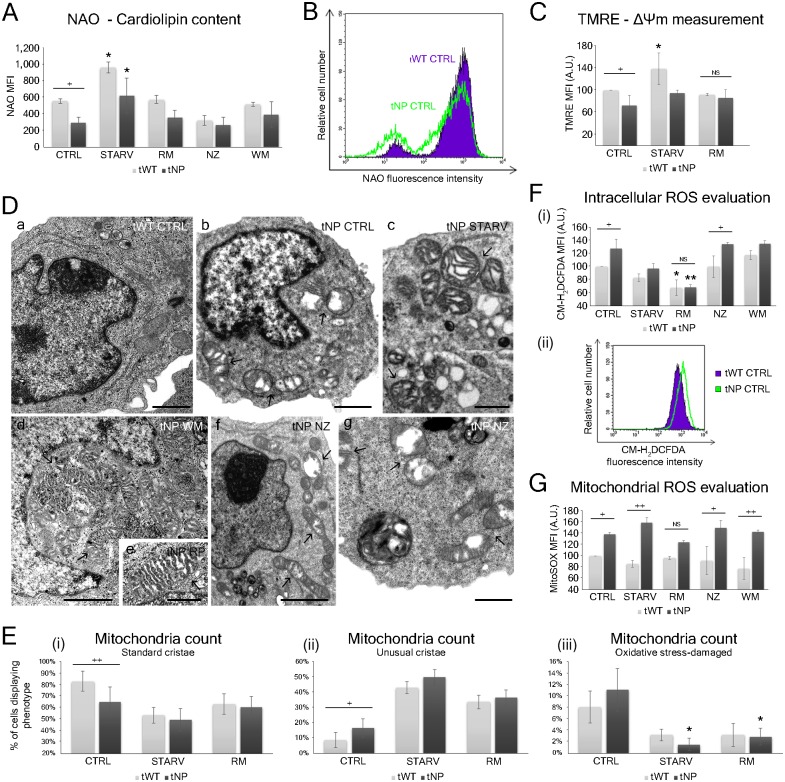
Evaluation of mitochondrial dysfunctional features by flow cytometry and transmission electron microscopy. (**A**) Statistical histogram of MFI variation of NAO in tWT and tNP cells for each experimental condition. Each value is expressed as a mean ± SD (Results from n ≥ 3 independent experiments); *P < 0.05 *vs* respective control. The difference between cell lines was significant as shown by two-way ANOVA (***P < 0.001). Two-way ANOVA with Bonferroni post test revealed a *P* value < 0.05 (^+^*P* < 0.05) between tWT and tNP in basal condition. (**B**) Flow cytometry histogram overlay depicting NAO MFI values of tWT and tNP basal condition. (**C**) Statistical histogram of MFI variation of TMRE in tWT and tNP cells for control, starved and rapamycin-treated cells. Mean values were converted to arbitrary units (A.U.) setting control values from wild-type cells as 100. Each value is expressed as a relative mean ± SD (Results from n ≥ 3 independent experiments); *P < 0.05 *vs* tWT control. The difference between cell lines was significant as shown by two-way ANOVA (*P < 0.05). Two-way ANOVA with Bonferroni post test revealed a *P* value < 0.05 (^+^*P* < 0.05) between tWT and tNP in basal condition. (**D**) Electron microscopy analyses of normal (**a**) and NP-B B lymphocytes (**b-g**) in control condition (**a**, **b**), and after starvation (**c**), rapamycin (**e**), nocodazole (**f**, **g**) and wortmannin (**d**) administration. In Niemann-Pick cells, dysfunctional mitochondria are recognizable (arrows): altered morphology is characterized by dilated and distorted cristae (**c**, **d, e**) and by stress-damaged features (**b**, **f**, **g**), absent in non-pathological cells (**a**). Bars: 1 μm for a, b, c, d, f, g; 0.5 μm for e. (**E**) Mitochondria from non-pathological and pathological cells were counted and classified according to their morphology—mitochondria with standard cristae (**i**), mitochondria with unusual cristae (**ii**) and oxidative stress-damaged mitochondria (**iii**)–in control, starved and rapamycin-treated cells. Each value is expressed as a percentage ± SD (n = 3; 50 cells counted/experiment); *P < 0.05 *vs* respective control. Two-way ANOVA with Bonferroni post test revealed a *P* value < 0.01 and < 0.05 for standard and unusual cristae numbers respectively (^++^*P* < 0.01 for standard cristae and ^+^*P* < 0.05 for unusual cristae) between tWT and tNP in basal condition. (**F**) ROS detection by CM-H_2_DCFDA in flow cytometry. (**i**) Statistical histogram of MFI expression of CM-H_2_DCFDA in tWT and tNP cells. Mean values were converted to arbitrary units (A.U.) setting control of wild-type cells as 100. Each value is expressed as a mean ± SD (Results from n ≥ 3 independent experiments); *P < 0.05 and **P < 0.01 *vs* respective control. Two-way ANOVA with Bonferroni post test revealed a *P* value < 0.05 (^+^*P* < 0.05) between tWT and tNP in basal condition and after nocodazole treatment; NS: not significant. (**ii**) CM-H_2_DCFDA flow cytometric histograms are overlaid to show the comparison between MFI values of tWT and tNP basal condition. (**G**) Statistical histogram of MFI expression of MitoSOX in tWT and tNP cells. Mean values were converted to arbitrary units (A.U.) setting as 100 control MFI values of wild-type cells. Each value is expressed as a mean ± SD (Results from n ≥ 3 independent experiments). Two-way ANOVA with Bonferroni post test revealed a *P* value < 0.05 (^+^*P* < 0.05) between tWT and tNP in basal condition and after nocodazole treatment, and a *P* value < 0.01 (^++^*P* < 0.01) between tWT and tNP after starvation and wortmannin treatments; NS: not significant.

We investigated the cardiolipin content using Nonyl Acridine Orange (NAO) (Fig 2A and 2B). This phospholipid has an important role in mitochondrial bioenergetic processes. Cardiolipin has been shown to interact with a number of inner mitochondria membrane proteins, including the respiratory chain complexes and substrate carriers, and it is involved in different stages of the mitochondrial apoptosis process as well as in mitochondrial membrane stability and dynamics. Fig 2A shows NAO Mean Fluorescence Intensity (MFI) values in Niemann-Pick and normal lymphocytes: in pathological samples, NAO MFI values were 40% lower than those measured in wild-type samples, as expected. This MFI reduction is consistently present in each experimental condition, if compared to wild-type samples, with a statistically significant (*P* < 0.001, two-way ANOVA) trend. This reduced intensity in fluorescence, observed also by means of another common fluorescent probe, MitoTracker Green (MTG; S1 Fig), confirmed the same trend traced by cardiolipin staining: altered mitochondria represent one of the main differences between the two cell models. Furthermore, mitochondrial membrane potential (ΔΨm), analyzed by means of TMRE, was significantly (*P* < 0.05, two-way ANOVA) lower in tNP control compared to tWT control lymphocytes (Fig 2C).

Furthermore, the highest NAO MFI values were expressed by starved cells from both cell lines, suggesting an increase in mitochondrial mass: the relative NAO intensity of starved cells was significantly higher than that of untreated cells (*P* < 0.05; Fig 2A), with a 1.7 and 2.2 fold increase for tWT and tNP cells respectively. We observed a similar increase in the relative intensity of other fluorescent dyes, MitoTracker Green (MTG) and MitoTracker Red (MTR) (S1 Fig). Moreover, cells showed a significant (*P < 0.05, two-way ANOVA) mitochondrial hyperpolarization in response to starvation (by means of TMRE; Fig 2C). These results show that nutrient deprivation leads to an increase in the mitochondria mass/number and in the mitochondrial ΔΨ in both cell lines. Indeed, nutrient starvation was shown to trigger a specific stress response called stress-induced mitochondrial hyperfusion [17], and to partially inhibit fission, a phenomenon able to protect mitochondria from autophagic catabolism when needed [18]. Furthermore, mitochondrial fusion occurs preferentially between mitochondria with higher ΔΨm [19]. These effects are consistent with a mitochondrial remodelling that help to maximize the capacity for oxidative phosphorylation under stressful conditions. In fact, when mitochondrial dynamics are disrupted, cellular dysfunction ensues.

Interestingly, rapamycin-treated samples displayed NAO and TMRE MFI values similar to the corresponding controls in both cell lines, revealing a general maintenance of mitochondrial status.

Moreover, TEM showed the presence of abnormal mitochondria mostly in NP samples (Fig 2D). Indeed, the Niemann-Pick type B cell line exhibited a deep alteration of mitochondrial morphology (in both basal condition and treated cells), which can be divided in two groups: **a**) swelled mitochondria with dilated and distorted cristae (defined as unusual); **b**) emptied and partially degraded mitochondria, typically caused by oxidative stress. A morphology-based mitochondria count (Fig 2E) revealed a higher content of normal mitochondria (with standard cristae) in basal wild-type lymphocytes compared to basal tNP cells (83% ± SD for tWT and 65% ± SD for tNP cells), with a high significance (**P < 0.01, two-way ANOVA). Autophagy stimulation (in particular starvation) induced a large increase in unusual mitochondria (with dilated and distorted cristae) in both cell lines and a concomitant reduction in oxidative stress-damaged mitochondria (*P < 0.05 for tNP *vs* control). Rapamycin, as well as starvation, elicited a 4±SD fold decrease in oxidative stress-damaged mitochondria in tNP cells, as well as a 2.6±SD fold reduction in tWT lymphocytes, pointing to a process of damaged mitochondria clearance common to both lines but more pronounced in pathologic cells. In addition, a large number of mitochondria were even detected within autophagic vacuoles, suggesting the presence of a composite mitophagic process (see following sections).

In addition, intracellular ROS levels, obtained by the CM-H_2_DCFDA probe (Fig 2F), increased in tNP lymphocytes compared to normal lymphocytes (*P < 0.05, two-way ANOVA). In agreement with TEM data, induction of autophagy reduced oxidative stress in both cell lines. In particular, rapamycin was the trigger inducing the maximum ROS decrease in both tWT and tNP cells, eliciting similar levels. However, this response to rapamycin was stronger in tNP cells (**P < 0.01 *vs* control), reducing ROS levels 2.1±SD fold compared to a 1.6±SD fold reduction in tWT (if compared to respective controls). Conversely, autophagy inhibitors led to intracellular ROS accumulation.

We also investigated mitochondrial ROS using MitoSOX Red (Fig 2G), observing their basal highest amount (corresponding to mitochondrial superoxide production) in pathological samples (*P < 0.05, two-way ANOVA). Once more, rapamycin was able to reduce mitochondrial ROS levels in tWT and, to a greater extent, in tNP samples.

### Autophagic vacuoles (AVs) and aberrant endocytic activity detection

**Fig 3:**
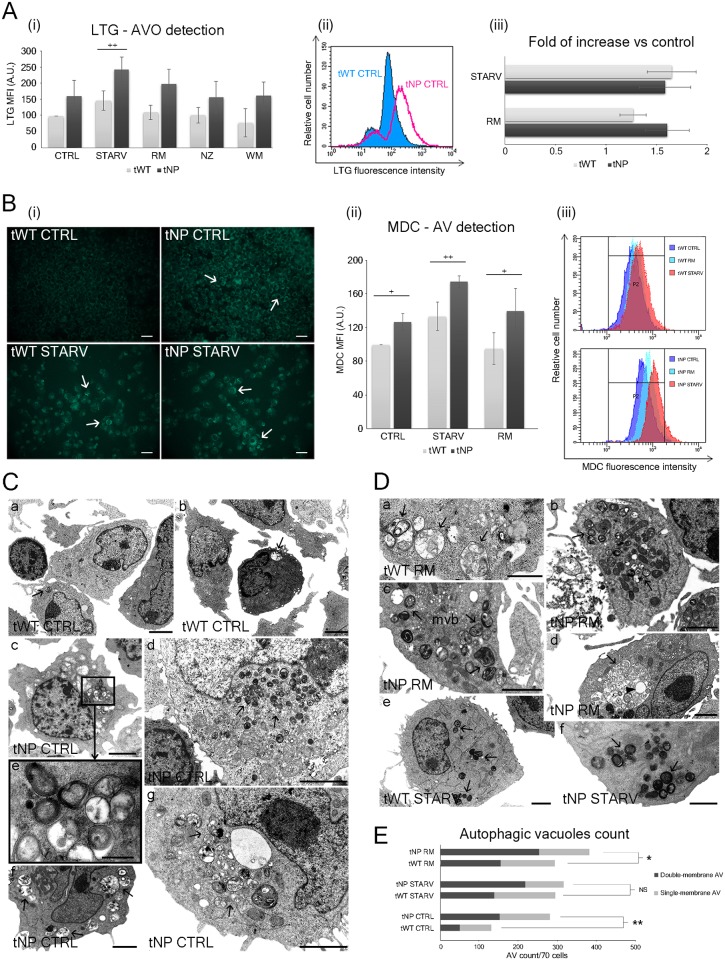
Autophagic Vacuole (AV) detection and endocytic compartment evaluation. (**A**) Acidic vesicular organelle (AVOs) detection by LysoTracker Green (LTG) in flow cytometry. (**i**) Statistical histogram of MFI variation of LTG in tWT and tNP cells for each experimental condition. Mean values were converted to arbitrary units (A.U.) setting control of wild-type cells as 100. Each value is expressed as a relative mean ± SD (Results from n ≥ 3 independent experiments). The difference between cell lines was determined to be significant by two-way ANOVA (***P < 0.001). Two-way ANOVA with Bonferroni post test revealed a *P* value < 0.01 (^++^*P* < 0.01) between tWT and tNP starved cells (**ii**) Flow cytometry histogram overlay showing lysotracker green MFI values of tWT and tNP basal condition. (**iii**) Fold increase related to histogram in (**i)** starved and rapamycin-treated cells in both cell lines, *versus* respective control. (**B**) Autophagic vacuole detection by Monodansylcadaverine (MDC) in fluorescence microscopy and flow cytometry. (**i**) Microscopy images showing monodansylcadaverine fluorescence from control and starved tWT and tNP cells. White arrows indicate cells rich in AV. Bars: 10μm. (**ii**) Statistical histogram depicting MFI variation of monodansylcadaverine in tWT and tNP cells for control, starved and rapamycin-treated cells. Mean values were converted to arbitrary units (A.U.) setting control of wild-type cells as 100. Each value is expressed as a relative mean ± SD (Results from n ≥ 3 independent experiments). Two-way ANOVA with Bonferroni post test revealed a *P* value < 0.05 (^+^*P* < 0.05) between tWT and tNP in basal condition and rapamycin treated and a *P* value < 0.01 (^++^*P* < 0.01) between tWT and tNP after starvation treatment (**iii**) Monodansylcadaverine flow cytometric histograms are overlaid to show the comparison among MFI values of basal condition, starved and rapamycin-treated samples in tWT (upper panel) and tNP (lower panel) lymphocytes. (**C**) TEM autophagic vacuole detection in control condition of tWT (**a**, **b**) and tNP (**c-g**) cells. Black arrows indicate AVs. Bars: 2 μm for a, b, c, d, f, g; 0.5 μm for e. (**D**) TEM autophagic vacuole detection in normal (**a**, **e**) and NP-B B lymphocytes (**b**, **c**, **d**, **f**), after nutrient deprivation (**e**, **f**) and rapamycin administration (**a-d**). Black arrows indicate AVs. Black arrowhead indicates a lipid droplet inside AV. Mvb: multivesicular bodies. Bars: 1 μm for a, f; 2 μm for b, c, d, e. (**E**) Double- and single- membrane autophagic vacuoles from tWT and tNP lymphocytes were counted in control, starvation and rapamycin conditions. Each value is expressed as an absolute number (70 cells counted/experiment). Two-way ANOVA with Bonferroni post test revealed a *P* value < 0.01 between tWT and tNP basal condition and a *P* value < 0.05 between tWT and tNP rapamycin treated.

To deeply characterize the autophagic pattern, we extended our investigation by employing the lysosomal and autophagic vacuole markers LysoTracker Green (LTG), Acridine Orange (AO) and Monodansylcadaverine (MDC). LTG and AO dyes are specific for the detection of acidic vesicular organelles (AVOs), represented predominantly by lysosomes and autolysosomes, whereas MDC prevalently accumulates in autophagosomes [20,21]. Cytometric and statistical histograms showed a LysoTracker Green (LTG) fluorescence intensity (Fig 3A and S1 Fig) that was greater in tNP cells than in their normal counterparts (a 1.65 fold increase ±SD), indicating a wider number of acidic vesicular organelles in the Niemann-Pick cell line. TEM images in Fig 3 also reveal a greater autophagic vacuole accumulation if compared to the accumulation occurring in normal cells. The increase in lysotracker green MFI persisted in each experimental condition in contrast to what occurred in wild-type samples, with a statistically significant (*P* < 0.001, two-way ANOVA) trend. Starved normal B lymphocytes showed a MFI increase for lysotracker green comparable to the increase observed in starved tNP cells, whereas pathological samples displayed a sharp increase for rapamycin-treated cells (WT and NP samples showing a 1.25 fold and 1.6 fold increase ±SD respectively) (Fig 3A). Nocodazole did not affect any of the two cell lines, whereas wortmannin inhibited tWT but not tNP, indicating an alteration of the phosphoinositide 3-kinase (PI3K) pathway in the latter cell line. Furthermore, we assessed the rate of acidic vesicular organelle accumulation using Acridine Orange (AO). Data from this acidotropic dye (S2 Fig) confirmed results obtained using lysotracker green. Indeed, both markers detected **i**) an increase in the number of acidic vesicular organelles in tNP cells, **ii**) the highest increase in pathological samples after rapamycin treatment and serum deprivation, and **iii**) a different wortmannin response in the two cell lines. These data were further corroborated by quantification of intracellular LAMP-1/CD107a, a major integral membrane glycoprotein of late endosomes and lysosomes (S2 Fig): slight differences (with the same trend observed for lysotracker green and acridine orange) appear among the different treatments, concomitantly highlighting clear and significant changes between the two cell lines (*P* < 0.001, two-way ANOVA). Once again we found that the tNP cell line basally presents the highest percentages of LAMP-3/CD63 positive events (S2 Fig), which also points to the impairment of endocytic trafficking.

Microscopic and cytometric analyses from monodansylcadaverine labelling (Fig 3B) focused on autophagic vacuole (AV) accumulation in tNP control cells (*P* < 0.05, two-way ANOVA) and the consequent large AV accumulation increase observed after nutrient deprivation. However, monodansylcadaverine can indicate increased degradative activity, but it cannot entirely describe macroautophagy because it also marks autophagic compartments after their fusion to acidic endo/lysosomes. To clearly visualize autophagosomes we paired the monodansylcadaverine protocol to LC3B-GFP staining, a well-characterized indicator of autophagosome formation, and we observed an increase in autophagosomes in tNP cells compared to wild-type cells (S2 Fig): tNP cells accumulated LC3B-positive puncta and had increased MFI for LC3B: following rapamycin treatment, we found an increase in the expression of LC3B in both cell lines.

TEM analyses confirmed the accumulation of autophagic vacuoles (single- and double-membrane) in tNP cells in the control condition (Fig 3C–3E) and after autophagy induction (both rapamycin and nutrient deprivation) (Fig 3D and 3E), concomitantly revealing the difference in the number and type of autophagic vacuoles between normal and pathologic cells. In particular, in tNP control cells we found a prevalence of complex double membrane vesicles (autophagosomes), usually localized near the nuclear membrane or, sometimes, close to emptied mitochondria. In both tNP and tWT cells, rapamycin and nutrient deprivation induced cytoplasmic autophagosome amassment. Particularly in tNP cells, such amassment is found close to mitochondria with aberrant cristae and, sometimes, in the presence of multivesicular bodies. Moreover, predominantly after starvation, whole mitochondria appeared inside autophagic vacuoles. Single-membrane (autolysosomes) and double-membrane vesicles (autophagosomes) were found to be equally represented in wild-type lymphocytes, basally and after both starvation and rapamycin administration (Fig 3C, 3D and 3E). These observations suggest a block of autophagy at the fusion step in tNP cells, since endolysosomes and autolysosomes are transient organelles under physiologic conditions.

### Detection of mitophagy and mitochondria-derived vesicles

The whole series of fluorescent probes for mitochondria revealed mitochondrial dysfunction and TEM showed the contemporary presence of abnormal mitochondria in tNP cells. Electron microscopy also highlighted lipid droplets and mitochondria characteristic for each cell line, with mitochondria-derived vesicle (MDV) formation—or Type 3 mitophagy (or micromitophagy) [22–24], whereas confocal microscopy revealed classic mitophagic features (Type 1-Type 2 mitophagy).

**Fig 4:**
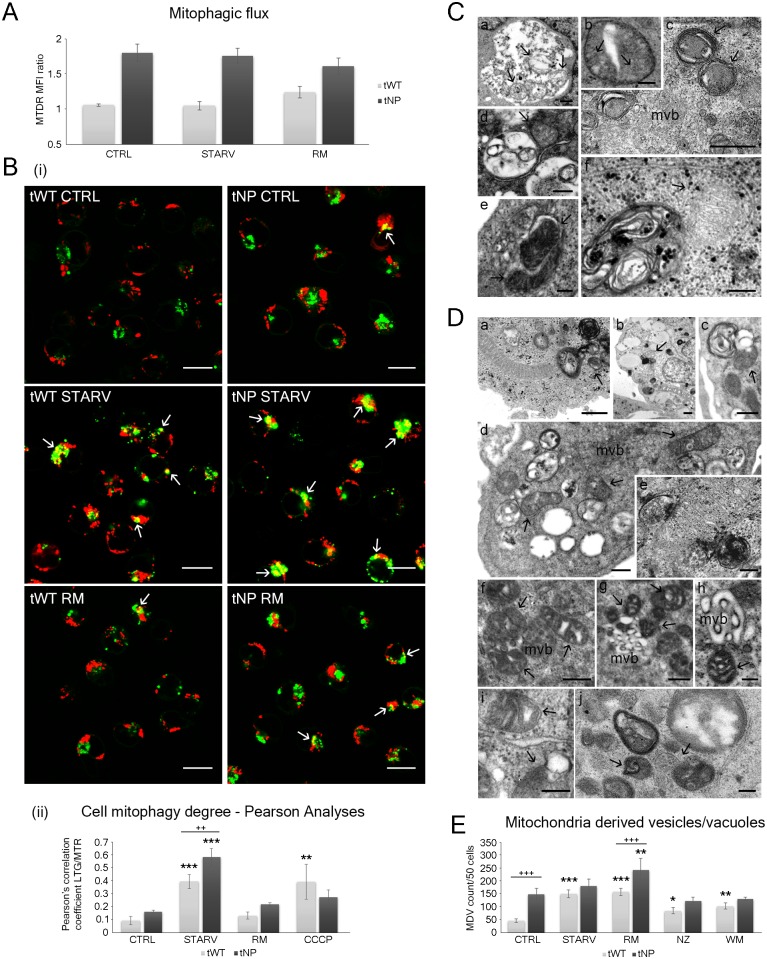
Mitochondrial autophagy evaluation by confocal and electron microscopy. (**A**) Mitophagic flux for control, starved and rapamycin-treated cells. For each experimental condition, MTDR fluorescence was measured in the presence or absence of chloroquine (CQ). Each value is expressed as the ratio of MTDR mean fluorescence intensity (MFI) in the presence of CQ to that in the absence of CQ. The difference between cell lines was determined to be significant by two-way ANOVA (***P < 0.001). (**B**) Evidence of mitophagy type 1/2 (classic mitophagy) in confocal microscopy. (**i**) Single confocal optical sections (~0.8 μm thickness) showing overlay of LTG (green) and MTR (red) in wild-type and pathologic samples for control, starved and rapamycin-treated cells. White arrows represent the LTG-labeled acidic organelles colocalizing with MTR-labeled mitochondria. Bars: 10 μm. (**ii**) Pearson's colocalization coefficient (PCC) of LTG and MTR for control, starved and rapamycin-treated cells. The uncoupling agent CCCP was used as a positive control. Pearson's coefficients were derived from three completely independent experiments with >10 fields per experiment contributing to the cumulative result. Each value is expressed as PCC ± SD; **P < 0.01 and ***P < 0.001 *vs* respective control. Two-way ANOVA with Bonferroni post test revealed a *P* value < 0.01 (^++^*P* < 0.01) between tWT and tNP starved cells. (**C**) TEM evidence of mitophagy type 1/2 (classic mitophagy) in Niemann-Pick cells in control condition (**a**), after nutrient deprivation (**b**, **c**) and rapamycin administration (**d**, **e**, **f**). In NP samples, mitochondria were recognizable within autophagic vacuoles (black arrows). Mvb: multivesicular bodies. Bars: 0.5 μm for a, c, d, f; 0.2 μm for b, e. (**D**) TEM evidence of mitophagy type 3 (micromitophagy) in normal (**a**, **i**) and NP-B B lymphocytes (**b-h**, **j**) after rapamycin administration (**a-h**) and nutrient deprivation (**i**, **j**). Black arrows indicate mitochondria-derived vesicles (MDVs) or mitochondria closely linked to autophagic vacuoles. Several of these structures were located right next to lipid droplets (**b**) and multivesicular bodies (mvb, **d**, **f-h**). Bars: 0.5 μm for a, b, d, f, g, i; 0.2 μm for c, e, h, j. (**E**) Mitochondria-derived vesicles (MDVs) from non-pathological and pathological cells were counted in all experimental conditions. Each value is expressed as an absolute number ± SD (n = 3; 50 cells counted/experiment). *P < 0.05, **P < 0.01 and ***P < 0.001 *vs* respective control. The difference between cell lines was determined to be significant by two-way ANOVA (***P < 0.001). Two-way ANOVA with Bonferroni post test revealed a *P* value < 0.001 (^+++^*P* < 0.001) between tWT and tNP basal condition and rapamycin-treated cells.

To better address mitophagic flux, we adopted the recent protocol established by Mauro-Lizcano and co-workers [25]: MTDR (MitoTracker Deep Red), applied in Flow Cytometry in combination with a selective inhibitor. Briefly, incubation with different mitophagy inducers resulted in a decrease in fluorescence, which was reversed in the presence of the lysosomal inhibitor chloroquine (see Material and Methods section). MTDR results, in Fig 4A, are related to both cell lines, in particular to control, starved and rapamycin-treated samples. The Niemann-Pick cell line immediately showed a physiologic mitophagic flux which was higher than reference lymphocytes (*P* < 0.05; two-way ANOVA), responding differently to serum deprivation and rapamycin: tWT lymphocytes showed the maximum “classic” mitophagy trend in rapamycin-treated samples, whereas the opposite was verified in rapamycin-treated tNP cells, with a mitophagy rate that was even lower than that which was observed in untreated lymphocytes.

To further investigate whether the classic mitophagy pathway in tNP cells was functional, lymphocytes were stained with LysoTracker Green and MitoTracker Red (Fig 4B). In addition, Pearson’s correlation coefficient (PCC) was used as a standard to measure colocalization between the green fluorescence of LysoTracker Green (LTG) and the red fluorescence of MitoTracker Red (MTR), indicating mitophagy (Fig 4B). Autophagic vacuole/mitochondrial colocalization was particularly evident already in control tNP cells, mostly after nutrient-deprivation, with a Pearson’s correlation coefficient of about 0.55±SD (*P* < 0.001). TEM observations provided additional information showing aberrant and degenerated mitochondria inside autophagic vacuoles (Fig 4C). These features, observed using both confocal microscopy and TEM, together with the low mitophagic flux found in starved and rapamycin-treated tNP cells, show that the progression of classic mitophagy is compromised in this cell line.

Interestingly, TEM analyses revealed the presence of Type 3 mitophagy, suggesting the existence of a composite/reparative mitophagic process (Fig 4D). Indeed, several mitochondria, particularly for rapamycin-treated tNP cells, appeared close to autophagic vacuoles or forming vesicles (MDVs). It has recently been reported that MDVs containing oxidized mitochondrial proteins bud off from mitochondria and then become internalized in multivesicular bodies (MVBs) [24,26]. Furthermore, multivesicular bodies appeared near these composite structures (Fig 4C and 4D) suggesting a subsequent fusion with lysosomes to complete hydrolytic degradation of the mitochondrial fragments. These data should be considered together with the evident reduction in ROS production (produced by rapamycin in tNP cells) and the limited activity of classic mitophagy in the same cells. We performed an absolute count of MDV features, highlighting the trend shown in Fig 4E. Rapamycin strongly promoted MDV production on both lines with high significance (*P* < 0.001 for tWT and *P* < 0.01 for tNP cells). Interestingly, Rapamycin succeeded in inducing MDV in tNP pathological cells, a normo- behavior, common to tWT lymphocytes. The same features did not appear after serum deprivation. Conversely, starvation stimulated MDV formation in tWT lymphocytes but seemed to privilege the classic mitophagic pattern in tNP cells (*P* < 0.001 for tWT and not significant for tNP cells). Both these systems aim to reduce the persistence of abnormal mitochondria, typical of many LSDs. This pathway is active under steady-state conditions and is further stimulated by oxidative stress. These mitochondria-derived vesicles may constitute a mechanism that selectively removes oxidized mitochondrial proteins while leaving the whole organelle intact. We can therefore consider rapamycin as a useful promoter of the clearance of damaged mitochondria. Moreover, it does not trigger apoptotic/necrotic events found in serum deprivation, which are potentially dangerous to the organism.

### Evaluation of cellular lipid content, lipophagy and lipid extrusion

**Fig 5:**
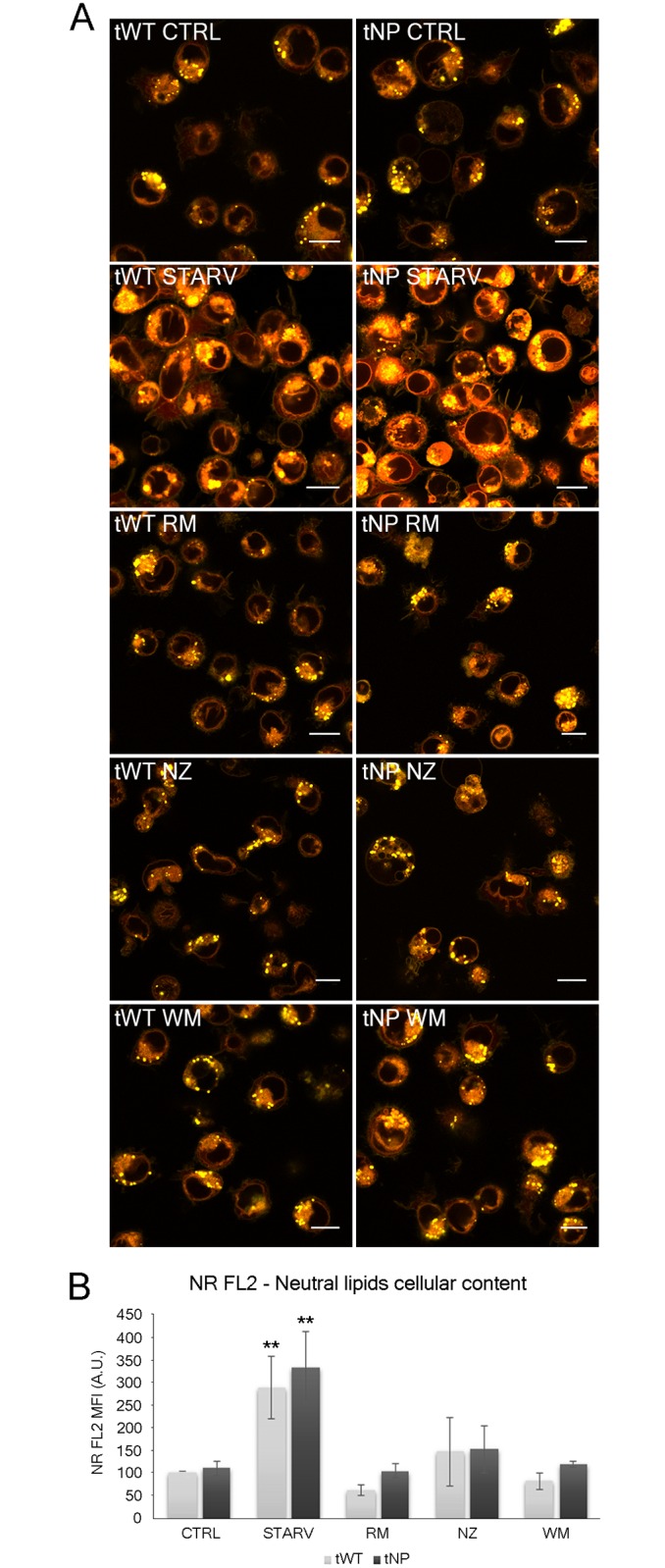
Evaluation of intracellular lipid content by Nile Red (NR). (**A**) Single confocal optical sections (~0.8 μm thickness) showing overlay of yellow (FL2) and red (FL3) NR fluorescence from all experimental conditions in tWT and tNP cells. Bars: 10 μm. (**B**) Statistical histogram of FL2 MFI variation of intracellular NR in tWT and tNP cells for each experimental condition. Mean values were converted to arbitrary units (A.U.) setting control of wild-type cells as 100. Each value is expressed as a relative mean ± SD (Results from n ≥ 3 independent experiments); **P < 0.01 *vs* respective control.

**Fig 6:**
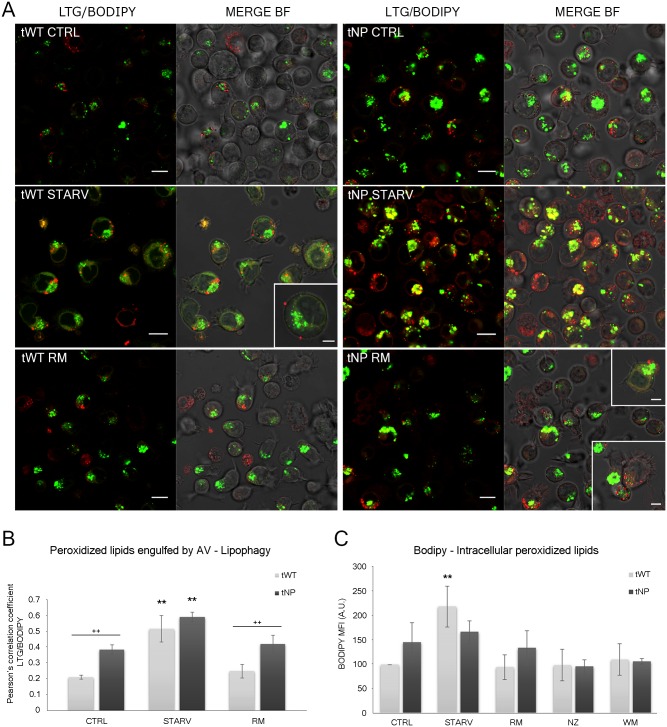
Evaluation of intracellular lipid content and lipophagy by BODIPY^®^ 665/676 (BP). (**A**) Single confocal optical sections (~0.8 μm thickness) showing overlay of LTG (green) and BP (red) and the relative merge with bright field (BF), from control, nutrient deprivation and rapamycin- treated tWT and tNP cells. Insets show lipid droplets bound to the outer cell surface, probably newly extruded from the cell, or just below the outer cell membrane, most likely about to be exocytosed by the cell. Bars: 10 μm; 5 μm for insets. (**B**) Pearson's colocalization coefficient (PCC) for LTG and BP in control and pathologic cells for control, nutrient deprivation and rapamycin administration. Pearson's coefficients were derived from three completely independent experiments with >10 fields per experiment contributing to the cumulative result. Each value is expressed as PCC ± SD; **P < 0.01 *vs* respective control. The difference between cell lines was determined to be significant by two-way ANOVA (***P < 0.001). Two-way ANOVA with Bonferroni post test revealed a *P* value < 0.01 (^++^*P* < 0.01) between tWT and tNP basal condition and rapamycin-treated cells. (**C**) Statistical histogram of MFI variation of intracellular BP in tWT and tNP cells for each experimental condition. Mean values were converted to arbitrary units (A.U.) setting control of wild-type cells as 100. Each value is expressed as a relative mean ± SD (Results from n ≥ 3 independent experiments); **P < 0.01 *vs* respective control.

To characterize lipid accumulation in Niemann-Pick cultured lymphocytes we employed the fluorescent dyes Nile Red (NR) and BODIPY^®^ 665/676 (BP), described in detail in the “Materials and Methods” section. In Figs 5 and 6, cytometric results show peculiar staining of both normal and pathologic cells. Briefly, NR dye emits on both FL2 (neutral lipids) and FL3 (polar lipids). Differences between the two emission wavelengths were detected by confocal microscopy (Fig 5A), where cytoplasmic lipid droplets appeared yellow-gold (FL2) and intra-/extra-cellular membranes appeared orange-red (FL3). In both cell lines, starvation promoted a sharp increase in yellow-gold inclusions (Fig 5A).

At the same time, using FC, we observed an NR MFI basal level that was slightly higher in tNP than in tWT cells and a considerable increase (*P* < 0.01) induced by nutrient deprivation in both cell lines for FL2 emission wavelengths (Fig 5B). Conversely, rapamycin elicited a reduction in lipid droplets probably promoting their degradation/extrusion.

Furthermore, the BP MFI basal level appeared higher (to a much greater extent than NR) in tNP than in tWT cells. This difference between NR and BP is consistent with data from mitochondria and ROS evaluations, since BODIPY^®^ 665/676 is a lipid marker with a high affinity for peroxidized neutral lipids, suggesting a generalized cellular stress. Flow cytometric BP quantifications showed starvation as the condition causing the greatest MFI of the peroxidized lipid sensor BODIPY^®^ 665/676 levels (Fig 6C), in both cell lines, although the greatest MFI was found in normal cells (*P* < 0.01). On the contrary, rapamycin, although able to prime autophagic flux, did not show the same damaging effect, yielding MFI values comparable to those of control cells or even lower (Fig 6C). Confocal analyses of BP staining, (red channel in Fig 6A) showed different lipid behaviors for the two cell lines, revealing lipid polarization and lipid extrusion, which was also confirmed by absolute counting of medium NR^+^ particles (see following section), after rapamycin and starvation treatments. This feature is particularly well marked in starved tWT cells, and in tNP rapamycin-treated samples (insets in Fig 6A). Double labelling by BP and LTG (Fig 6A) allowed us to monitor colocalization of both of these markers, described by Pearson’s correlation coefficient (Fig 6B) and suggestive of lipophagy. Indeed, confocal images revealed an increase in peroxidized lipids engulfed by autophagic vacuoles following autophagy stimulation. Moreover, electron microscopy revealed numerous lipid droplets with monophasic density, particularly in cells from NP samples, similar to those recognized by Mochizuki et al. in bronchial and pulmonary tissues from sarcoidosis patients [27]. Some of these droplets were closely associated with mitochondria and were attached to the mitochondrial membrane, as described previously: lipid droplets, surrounded by numerous lysosome granules, were closely associated with swollen and cristae disorganized mitochondria membrane structures (see Fig 4Db). Additionally, some lipid droplets were found inside autophagic vacuoles (see Fig 3Dd) confirming lipophagic features. Multivesicular bodies (MVBs) were occasionally located near the lipid droplets.

Interestingly, it was found that in tNP cells, rapamycin, although not increasing BP positivity compared to untreated cells (Peroxidized Lipid Content ratio RM/CTRL: 0.94±SD), increased the degree of colocalization (Pearson’s Coefficient ratio RM/CTRL: 1.25±SD), whereas in tWT cells, these ratios were 1.25±SD and 1.3±SD respectively. These findings show that rapamycin has the ability to clear preexisting peroxidized lipids without triggering additive oxidative stress.

### Detection of microvesicles and lipid particles in extracellular environment—comparison with lipid cellular content

In order to characterize microvesicle/microparticle subpopulations we used different fluorescent lipid probes to label distinct cell compartments and/or microvesicle membranes.

We then assessed the lipid labelling patterns of both Nile Red (NR) and BODIPY^®^ 665/676 (BP), probes highlighting different steps of intracellular trafficking of lipid droplets. To this end, we first labelled cells in order to perform intracellular analyses by confocal microscopy and flow cytometry, as previously shown (see Fig 5), and then focused on particle content in medium using the flow cytometric logarithmic approach.

**Fig 7:**
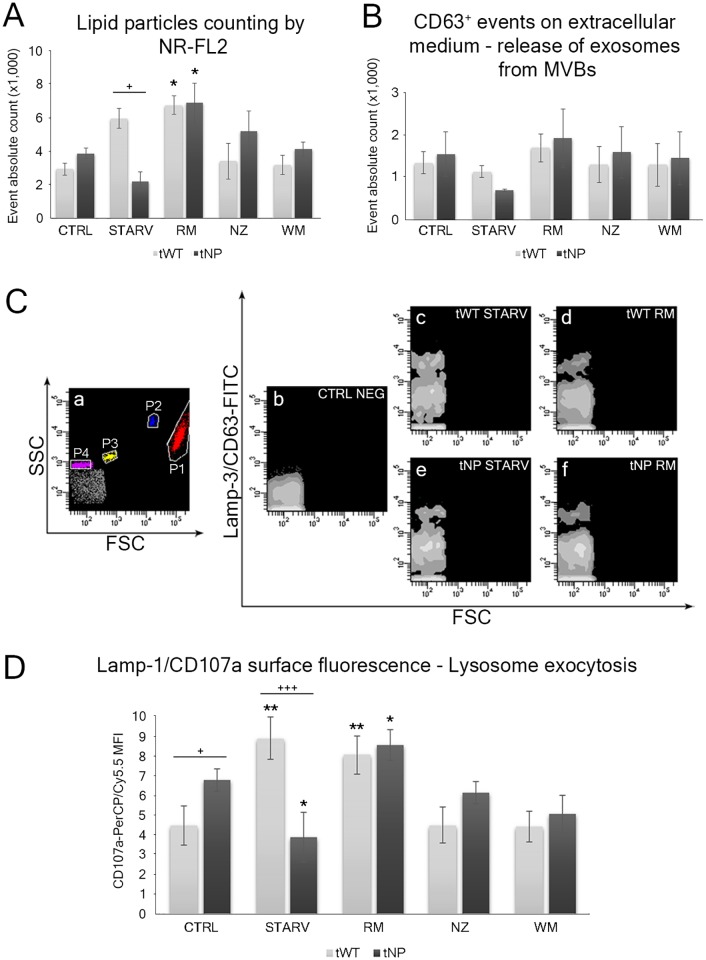
Detection of microvesicles and lipid particles in the extracellular environment and analysis of lysosomal exocytosis. (**A**) Statistical histogram showing lipid particle counting by NR-FL2 in the extracellular environment for each experimental condition. Each value is expressed as an absolute number ± SD (Results from n ≥ 3 independent experiments); *P < 0.05 *vs* respective control. Two-way ANOVA with Bonferroni post test revealed a *P* value < 0.05 (^+^*P* < 0.05) between tWT and tNP starved cells. (**B**) Statistical histogram showing CD63^+^ events counted in the extracellular environment for each experimental condition. Each value is expressed as an absolute number ± SD (Results from n ≥ 3 independent experiments). (**C**) Extracellular microparticle/microvesicle detection by FC. (**a**) Dot plot FSC *vs* SSC in logarithmic visualization. P1: viable cells; P2: Dako CytoCount counting beads 5.2 μm diameter; P3: beads 1 μm diameter; P4: beads 0.5 μm diameter; (**b**) Density plot FSC *vs* FL1 of the negative sample; (**c**) Density plot FSC *vs* FL1-CD63 of starved and (**d**) rapamycin-treated tWT cells. (**e**) Density plot FSC *vs* FL1-CD63 of starved and (**f**) rapamycin-treated tNP cells. (**D**) Statistical histogram of surface Lamp-1/CD107a detection on fresh cells: MFI values shown are for each experimental condition and from both cell lines. Each value is expressed as a mean ± SD (Results from n ≥ 3 independent experiments); *P < 0.05 and **P < 0.01 *vs* respective control. Two-way ANOVA with Bonferroni post test revealed a *P* value < 0.05 (^+^*P* < 0.05) between tWT and tNP basal condition and a *P* value < 0.001 (^+++^*P* < 0.001) between tWT and tNP starved cells.

We considered the output FL2 fluorescence of NR as lipid droplets. The histogram in Fig 7A shows, for tNP cells, a strong increase in NR FL2^+^ particle counts for rapamycin, followed by nocodazole and wortmannin to a lesser extent, whereas tWT cells show this increase (for NR FL2 particle counts) in starvation and with rapamycin treatment, as expected. It has been shown that microvesicles/microparticles (particularly exosomes) carry a whole set of fatty acids, which could be produced in the vesicle itself or loaded into exosomes/microvesicles during their biogenesis in the parental cell [28]. Our findings indicate that microparticle release is particularly primed by rapamycin in both cell lines. Furthermore, we suggest that the secretion of microvesicles and lipid particles contributes to the maintenance of cellular lipid homeostasis, altered in NP-B B lymphocytes. In fact, as previously discussed, peroxidized lipid particles appear as BP fluorescent particles on the extracellular surface of starved and rapamycin-treated cells (insets in Fig 6A). Lipid particle release may partially bypass the traffic block giving rise to the lysosomal lipid accumulation in cells from the Niemann-Pick type B cell line.

CD63 labelling was also performed. The statistical histogram in Fig 7B and cytometric histograms in Fig 7C reveal endosome escape ability from tWT and tNP cells, showing that starvation and rapamycin treatments act differently on tNP and tWT lymphocytes. Niemann-Pick cells show a noteworthy increase in CD63 positive particles in rapamycin-treated cells, whereas, such CD63 positive particles are less numerous in starved cells.

### Exocytosis detection: clearance of lysosomal lipid storage?

Discrepancies between particle counts from the two cell lines could be partially explained by CD107a staining of unfixed samples. This assay is able to trace the lysosome exocytosis shown in Fig 7D.

Firstly, it is noteworthy that this process appears to be different in tNP and tWT lymphocytes. Niemann-Pick cells show physiologically stronger and more extensive exocytosis processes than their normal tWT counterparts (*P < 0.05, two-way ANOVA). In pathologic cells, wortmannin administration induces a decrease in lysosomal exocytosis: this phenomenon is also observed in starved cells with a trend which is the opposite of that which is observed in tWT starved cells (***P < 0.001, two-way ANOVA). It is striking that rapamycin is able to conform the aberrant tNP response to the tWT normal reaction (reaching similar CD107a fluorescence values), acting as a homogeneous inducer of lysosomal exocytosis and a modulator of both the autophagy and exocytosis processes. Furthermore, data on lipid cellular content, lipid particles and lysosomal exocytosis, pooled together, suggest that lysosomes (and/or autolysosomes) may “dump” indigested lipids into the extracellular space, reducing lipid accumulation in the lysosome.

## Discussion

As mentioned above, LSDs are characterized by autophagy impairment, which also takes place in neurodegenerative diseases such as Alzheimer’s, Parkinson’s, Huntington’s, and Niemann-Pick [6,29–31]. Recently, tremendous progress has been made in characterizing the autophagy protein machinery and signalling cascades, resulting in an explosion of applied research in autophagy [32]. The discovery of mTOR and the understanding of its biological functions were greatly facilitated by the use of rapamycin, which inhibits some of the functions of mTOR [33,34].

Autophagy dysfunction is defined as excessive autophagy induction or blockade of autophagy flux, and it is recognized as a potential mechanism of cell death, resulting in either apoptosis or autophagic cell death (also referred to as type II programmed cell death) [35,36]. However, since the majority of data supports autophagy as a pro-survival pathway rather than a cell death pathway (autophagic paradox), with evidence of its possible direct role in cell death only coming from artificial systems in which apoptosis is chemically or genetically suppressed, the role of autophagy in “programmed cell death” remains debatable [35].

Niemann-Pick disease (NPD) type A and B are lysosomal storage diseases (LSDs) caused by the lack of acid sphingomyelinase (ASMase) activity [1]. The enzyme defect results in a pathological accumulation of sphingomyelin within lysosomes. In many LSDs, an accumulation of undegraded substrates in lysosomes, due to a deficiency of specific lysosomal enzymes, impairs the autophagic process [37].

First of all, our data show an increase in the lysosomal network in the tNP cell line and its concomitant impairment, which is consistent with what has been reported by other authors [5]. We also found that basally, NP-B B lymphocytes have an atypical autophagy pattern, with a considerable number of autophagic vacuoles. Treatments with specific inducers and inhibitors underline various aspects of this atypical autophagic pattern. Generally, an increase in autophagic vacuole content may reflect both an increase in autophagic flux (hyperactivation of autophagy) and an impairment of its late phases (block of autophagosome/lysosome fusion). In both conditions, the accumulation of autophagic vacuoles indicates “autophagic stress”. The hyperactivation of the lysosomal acidic system represents a cellular response protecting against product accumulation [38,39].

Autophagy impairment in Niemann-Pick cells has also been observed by Gabandé-Rodríguez and colleagues, who characterized an inefficient autophagolysosomal clearance in both NP-A fibroblast and neurons [5]. However, our study is the first to report the accumulation of partially degraded mitochondrial fragments and an atypical mitophagy pattern in Niemann-Pick type B-derived cells. Indeed, we revealed (by TEM and FC) mitochondrial abnormalities in tNP cells which are affected differently by different pharmacological treatments.

Under normal conditions, autophagy is kept at basal level to maintain cellular homeostasis and to preserve cell integrity by eliminating long-lived, overproduced, and aggregation-prone proteins or dysfunctional organelles such as damaged mitochondria [40]. Mitochondria are rich sources of proteins and lipids, and during nutrient deprivation, it is likely that autophagic events for survival involve mitochondria [10,41–44]. A proper balance of mitophagy and mitochondrial biogenesis seems essential to cellular well-being since inadequate and excess mitophagy can promote both cell injury and death [3,45–47]. In this cell model, we found an impaired mitophagic flux, autophagic vacuoles with mitochondria accumulated and concurrently unable to completely remove these organelles. Using a multiparametric approach, we found different forms of mitophagy: type 1, type 2 and type 3 or micromitophagy. The latter is characterized by the formation of mitochondria-derived vesicles (MDVs), cargo-selective vesicles released from mitochondria independently of the mitochondrial fission machinery. Oxidative stress may prime MDV formation, and the MDVs themselves are enriched in oxidized mitochondrial proteins [48]. We observed (by means of TEM) that this type of mitophagy was more pronounced in rapamycin-treated Niemann-Pick-derived cells. In fact, to prevent cellular damage by preserving a population of healthy mitochondria, several quality control mechanisms have evolved: the mitochondrial-derived vesicles may indeed constitute a mechanism that selectively removes oxidized mitochondrial proteins while leaving the whole organelle intact. Furthermore, autophagosomes were transiently located in proximity to the outer mitochondrial membrane and, in such cases, the structures were clearly connected. On the contrary, type 1/type 2 mitophagy was the prevailing mitophagic process represented in nutrient-deprived lymphocytes for oxidized mitochondria clearance. Mitochondria continually change shape through the combined actions of fission, fusion, and movement along cytoskeletal tracks [11]. Unusual and particularly oxidized cristae (detected and counted in both treated and untreated tWT and tNP cells; see Fig 2E) signal just this status, particularly evident in NP lymphocytes. This suggests that both autophagy inducers stimulate mitochondrial cristae remodelling which leads to the removal of damaged mitochondria by autophagy, as described by Zick and colleagues [49], although with different contributions from the two pathways: classic mitophagy and MDV formation. The persistence of such impaired mitochondria may cause their elimination through mitophagy, whereas MDVs are typical of an early, milder and more organized response of the mitochondrial network to stress. Our data point to rapamycin as an inducer of “milder and better arranged degradation” of damaged mitochondria: this process seems to be preferred by rapamycin-treated cells to reach mitochondrial network homeostasis.

**Fig 8:**
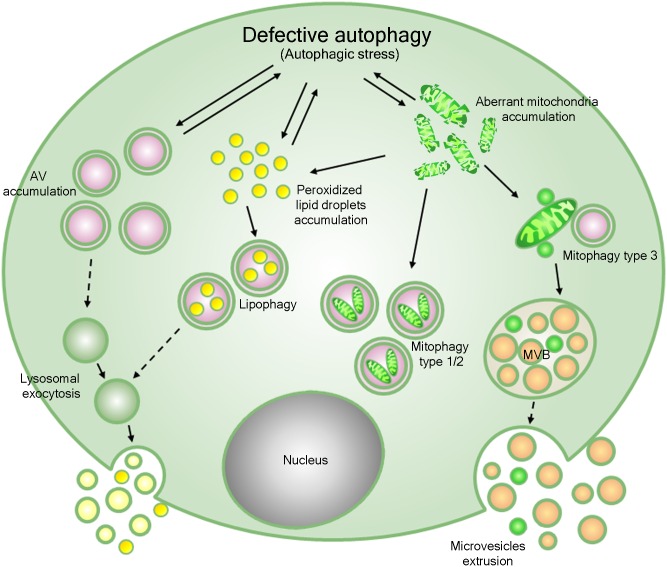
A proposed model for the pathogenesis of Niemann-Pick disease type B. Defective ASMase and lysosomal storage could lead to a reduced ability of lysosomes to fuse with autophagosomes. This in turn could result in a partial block of autophagy maturation and defective degradation, accompanied by accumulation of autophagic vacuoles, peroxidized lipid droplets and aberrant mitochondria (autophagic stress). Additional evidence and hypotheses are more fully explained in the text. Dotted lines: proposed mechanism; solid lines: proven mechanism.

We found that human NP-B B lymphocytes display considerable alteration in autophagic vacuole accumulation and mitochondrial dysfunction, also showing mitophagy induction. Lysosomal enzymatic clearance by known classic routes is clearly impaired in these cells; consequently, an imbalance between flux and clearance through the autophagic pathway appears, probably contributing to the clinical manifestation of Niemann-Pick disease type B (Fig 8). In NP-B B lymphocytes, evidence points to autophagy dysfunction (autophagic stress) in both the initial and final phases, with accumulation of altered mitochondria, features of classic mitophagy and MDV formation, enhanced ROS level, a dramatic increase in autophagic vacuoles and peroxidized lipid droplet amassment. Starvation and rapamycin treatment, by priming autophagy, deeply modulated these features.

Detection of several lipid droplets (also in relationship with mitochondria) [50–52] and MVBs by TEM analyses prompted us to investigate intracellular lipids and microparticle/microvesicle release. Intracellular lipid detection showed an accumulation of lipids (traced by CM and TEM) and evidence of lipophagy. We mainly discriminated between NR^+^ microparticles, virtually representing extruded lipids and CD63^+^ microvesicles. In rapamycin-treated cells, we observed an increase in the release of both types of vesicles. These data are consistent with data reported in exocytosis investigations and depict a scenario in which autophagy inducing conditions (particularly rapamycin administration) have a specific role in unconventional secretion while at the same time pointing to rapamycin as a potential therapeutic agent [53]. The exocytosis of lysosomes is considered to be important in the rapid resealing of plasma membrane injuries and also in microbiological defence mechanisms [54,55]. In the resealing process, the lysosomal membranes are integrated into the plasma membrane and thus serve as an intracellular membrane reservoir. Hence, this action helps to preserve cellular viability, a fundamental pre-requisite for pharmacological activity. Indeed, while starvation, as expected, induced a certain degree of cell death and a massive and dysregulated mitophagy, rapamycin was able to reduce intracellular and mitochondrial ROS, regulate MDV formation and lipid droplet amassment and thus maintain cell viability, prospecting its use as a pharmacologic agent as it is used in the treatment of other LSDs and neurodegenerative disorders [56,57]. The analyses of the intra and extra-cellular environments showed aberrant lipid accumulation in NP-B B lymphocytes, culminating in lipid microparticle extrusion (by lysosomal exocytosis mechanisms) or lipophagy. Rapamycin partially alleviated these pathological features by promoting MDV formation (mitophagy type 3), reducing lipid droplet amassment, microvesicle release and lysosomal exocytosis. In conclusion, all the features in this cell line show a deep autophagy/mitophagy alteration, revealing physiologic autophagic stress and defective mitochondrial clearance. Pharmacological activation of autophagy by rapamycin suggests that this agent may have the ability to modulate autophagy imbalance (at least in part) and hence could be used in supporting therapeutic schemes.

## Materials and Methods

### Cell culture

B lymphocyte cell lines derived from NP type B patient (cell line GM16193 EBV-transformed B cells deficient in the enzyme acid sphingomyelinase) and their respective normal counterparts (cell line GM22647 B lymphocytes EBV-transformed from healthy donor) were purchased from the Coriell Cell Repositories. Lymphoid cells were grown in a humidified 5% CO_2_ atmosphere at 37°C in RPMI 1640 medium (Sigma-Aldrich) containing L-glutamine, penicillin and 15% of fetal calf serum (FCS).

### Cell treatments

We evaluated different steps of the autophagic process using specific inducer/inhibitors, in particular, Rapamycin (RM; Sigma-Aldrich), Wortmannin (WM; Sigma-Aldrich) and Nocodazole (NZ; Sigma-Aldrich).

Rapamycin (RM), an antibiotic discovered as an anti-fungal agent in the 1970s, is an efficient inhibitor of mTOR resulting in autophagic induction [58]. In mammals, mTOR is the central player in autophagy signalling. It is a serine/threonine kinase which inhibits autophagy by controlling both translation and transcription of Atg proteins (autophagy-related proteins), therefore interfering with the formation of autophagosomes. Rapamycin and its analogues bind to a domain separate from the catalytic site to block a subset of mTOR functions. These drugs are extremely selective for mTOR and are already used in the clinic to treat cancer, but they could potentially activate an mTOR-dependent survival pathway that could lead to treatment failure.

Wortmannin (WM) has an autophagy-suppressive effect inhibiting class III PI3K (Phosphoinositide 3-kinase) complex. This complex is involved in the early stages of autophagosome formation, promoting the nucleation of autophagic vesicles [59].

Finally, we employed Nocodazole (NZ), an anti-neoplastic agent which exerts its effect in cells by interfering with the polymerization of microtubules, since autophagosome formation and the fusion of autophagosomes/lysosomes require an intact microtubule network [60].

Cells were pre-treated with rapamycin 100nM (24h before cell analysis), nocodazole 10μg/ml and wortmannin 100nM (pre-treatment of 2h).

To further induce autophagy we also grew cells in nutrient-deprived conditions (starvation): cells were incubated for 2h in glutamine-free medium without FCS and any other supplements (starvation medium).

### Flow cytometric detection of cell death

Cell death features (early and late apoptotic, as well as necrotic cells) were evaluated using two cytometric procedures: fluorescein isothiocyanate-conjugated Annexin V (FITC Annex-V; Immunostep) and supravital Propidium Iodide (PI; Sigma-Aldrich) stainings. Annex-V fluorescence is a standard method for measuring the amount of phosphatidylserine exposed on the outer face of the plasma membrane, following caspase activation, as an indicator of intermediate events of apoptosis. Fluorescence of the nucleic acid dye PI is a measure of the most advanced stages of apoptosis and even necrosis, since it is not permeant to live cells. Briefly, cells were resuspended in binding buffer (1x) and stained with Annex-V FITC according to the manufacturer’s instructions, or they were incubated 30 min in the dark with 50μg/ml PI. Cells were washed with phosphate-buffered saline (PBS) and then analyzed by flow cytometry (FC). Sample acquisition was performed by means of FACSCalibur flow cytometer equipped with CellQuest^™^ software. For each experimental condition, at least 10,000 total events were collected. Apoptotic and necrotic cells were detected as PI^dim^ and PI^bright^ clusters, respectively. Apoptotic cells were further identified as Anx^+^ population. An identical analysis was conducted on both tNP and control cells.

### Acidic compartment detection

Autophagic features can be monitored by quantifying the extension of acidic vesicular organelles (AVOs) by staining with acidotropic dyes that mark lysosomes and late autophagic vacuoles (autophagolysosomes and amphisomes) [61,62]. This analysis was performed using LysoTracker Green (LTG; Molecular Probes) and the weak base Acridine Orange (AO; Sigma-Aldrich) with both flow cytometry and confocal microscopy. These acidotropic probes are used as indicators of lysosomal stability/function in addition to quantification of the acidic compartment.

#### AO-uptake

The pH-sensitive dye Acridine Orange (AO) is widely used to detect acidic vesicular organelle formation [63,64]. AO is a cell-permeable fluorescent dye that, at its highest concentrations, stains DNA red and cytoplasm bright green. It can also enter acidic compartments, such as lysosomes and autolysosomes, where it becomes protonated and sequestered. At its lowest concentrations, in an acid environment, AO emits red fluorescence with an intensity proportional to the degree of acidity and/or to the acidic compartment volume [65]. Therefore, acidic vesicular organelle formation in AO-stained cells can be measured by flow cytometry or, alternatively, observed under a fluorescence microscope. Following treatment with inducers/inhibitors, cells were washed and resuspended in 0.5 ml RPMI, then stained with Acridine Orange 75ng/ml for 15 minutes at 37°C. Red lysosomal and green cytoplasmic fluorescence of 10,000 cells per sample were acquired by flow cytometry using the FL3 and FL1 channels respectively.

#### LysoTracker Green-uptake

LysoTracker Green (LTG) is a fluorescent probe used for the determination and localization of acid organelles in viable cells. It consists of a fluorophore linked to a weak base, partially protonated at neutral pH, permeable to cell membranes and typically tending to concentrate in spherical organelles. LTG fluorescence measured by cytometry represents the overall mass of acidic organelles and reflects autophagic induction [66]. The acidotropic dye LysoTracker Green was diluted in RPMI. Cells were cultured at 37°C and resuspended in prewarmed (37°C) medium containing 50nM LysoTracker Green for 45 min [64]. Cells were then resuspended in fresh pre-warmed medium, and green lysosomal fluorescence of 10,000 cells per sample was determined by flow cytometry using the FL1 channel. CellQuest^™^ software was used to analyze all of the data from flow cytometric experiments.

### Autophagy detection

Autophagy in living cells was detected in flow cytometry and fluorescence microscopy by monodansylcadaverine (MDC) staining and by monitoring the cellular distribution of GFP-LC3BII using GFP-LC3B.

#### Monodansylcadaverine staining

The fluorescent drug Monodansylcadaverine (MDC; Sigma-Aldrich) was used as a tracer for autophagic vacuoles (AVs). MDC is a specific in vivo marker that preferentially accumulates in autophagic vacuoles due to a combination of ion trapping and specific interactions with membrane lipids [67,68]. Following induction/inhibition of autophagy, the cells were incubated with 50μM MDC in PBS at 37°C for 10 minutes. To visualize the MDC blue fluorescence emission, we used a Nikon TS100 fluorescence microscope with UV (338nm) excitation light. MDC labelled lymphocytes were also acquired by means of FACSCanto II flow cytometry, collecting at least 10,000 events for each different sample, in order to measure autophagic flux [25,68].

#### LC3B protein aggregation

Autophagy was also detected by measuring the aggregation of LC3B protein coupled to green fluorescence protein (GFP) using the Premo Autophagy Sensor Kit (Molecular Probes), according to the guidelines. Briefly, tWT and tNP cells were transduced with BacMam LC3B-GFP with a multiplicity of infection (MOI) equal to 30, using 6×10^4^ cells in 96-multiwell plates. Mutated LC3B(G120A)-GFP was used as a negative control. Twenty-four hours after transduction, cells were treated with rapamycin (100nM) for 24h. The appearance of LC3B-GFP aggregates was observed and photographed using a confocal microscope [69]. Quantitative analyses of LC3B-GFP mean fluorescence intensity were performed by ImageJ software. Image analyses were carried out by selecting the cells in the images and determining the mean fluorescence intensity of all the pixels selected. Subsequently, the relative fluorescence intensity was calculated by setting the mean fluorescence intensity of the first image in a time series to 100%.

### Determination of mitochondrial integrity and functions

The cardiolipin-sensitive probe Nonyl Acridine Orange (NAO; Sigma-Aldrich) is able to monitor changes in mitochondrial lipids [64,70,71]. NAO, used at low concentrations in living cells, is an efficient fluorescent indicator for cardiolipin of inner mitochondrial membranes. In the present study, it was used to measure the mitochondria mass/number independently of mitochondrial membrane potential (ΔΨm). After an incubation of cells with 100nM NAO for 15 min at 37°C in the dark, samples were acquired by flow cytometry using the appropriate fluorescence channels. Mitochondria were also stained with MitoTracker Green (MTG) 100nM for 30 minutes at 37°C. Cells were then analysed by flow cytometry, collecting at least 10,000 events for each sample.

The ΔΨm was analysed using the (ΔΨm)-specific stain TMRE, a tetramethylrhodamine able to selectively enter into mitochondria according to the Nernst equation [72,73]. Cells were loaded with 40 nM TMRE at 37°C for 15 min and the analysis was carried out under nonquenching conditions. The specificity of this analysis was determined using the uncoupling agent CCCP (carbonyl cyanide m-chlorophenylhydrazone; Sigma-Aldrich), which caused mitochondrial membrane depolarization with a sudden decrease of TMRE fluorescence. Cells were treated for 30 min with 10μM CCCP, stained with TMRE as described previously and then acquired by flow cytometry. Fluorescence intensity is expressed as the initial signal after background subtraction.

### Flow cytometric evaluation of cellular and mitochondrial ROS

Reactive oxygen species generation was determined by flow cytometry after cells were stained with CM-H_2_DCFDA (Molecular Probes), which specifically detect the generation of intracellular H_2_O_2_. Samples of both cell lines were analyzed as fresh cells for the CM-H_2_DCFDA test. The fluorescent probe 5-(and-6)-chloromethyl-2′,7′-dichlorodihydrofluorescein diacetate acetyl ester (CM-H_2_DCFDA) is a cell membrane permeable compound and is converted into the cell membrane impermeable nonfluorescent compound, H_2_DCF, by intracellular esterases. Oxidation of H_2_DCF by hydrogen peroxide produces the highly fluorescent DCF (dichlorofluorescein). The fluorescence intensity of DCF inside the cells is proportional to the amount of peroxide produced. CM-H_2_DCFDA was solubilized in dimethyl sulfoxide (DMSO; Sigma-Aldrich) and then diluted to a final concentration of 5μM in PBS cellular pellets for 30 min at 37°C [74]. Cells were then washed once with PBS and were acquired by flow cytometry, collecting at least 10,000 events for each different sample. CM-H_2_DCFDA fluorescence intensity is expressed as the initial signal after background subtraction.

The production of ROS by mitochondria was analysed in flow cytometry using MitoSOX Red (Molecular Probes). This reagent is a fluorogenic dye specifically targeted to mitochondria in live cells. Oxidation of this probe by superoxide produces red fluorescence. MitoSOX 5μM was added to the samples 10 min before the time of acquisition. Sample were analysed by flow cytometry using the appropriate fluorescence channel. MitoSOX fluorescence intensity is expressed as the initial signal after background subtraction.

The accuracy of ROS analysis was determined using the oxygen free radical scavenger N-acetylcysteine (NAC; Sigma-Aldrich) as a negative control: cells were treated for 24h with 5mM NAC, stained with CM-H_2_DCFDA or MitoSOX as described previously and then acquired by flow cytometry.

### Monitoring of mitochondrial autophagy (mitophagy) and mitophagic flux

In order to visualize mitochondrial entrapment in autophagic vacuoles and, accordingly, monitor different steps of mitophagy we performed a co-labeling with LysoTracker Green (LTG) and the mitochondrial probe MitoTracker Red CMXRos (MTR; Molecular Probes) [66]. MTR passively diffuse across the plasma membrane of live cells and accumulate in active mitochondria [75]. For confocal live imaging, cells were grown on MatTek glass bottom chambers and stained with LTG 200nM for 45 minutes at 37°C. During the last 15 min of incubation, cells were exposed to MTR 200nM, re-cultured at 37°C for 15 minutes and then analyzed by a Leica TCS SP5 II confocal microscope. Colocalization analyses (Pearson's correlation coefficient) were performed using JACoP plugin in ImageJ [76]. Cells treated with the membrane uncoupler and mitophagy inducer CCCP were used as a positive control for colocalization analyses. Cells were treated for 30 min with 10μM CCCP, stained with LTG/MTR, as described previously, and then observed under a confocal microscope. LTG/MTR double marked lymphocytes were also acquired by flow cytometry, collecting at least 10,000 events for each different sample. Furthermore, transmission electron microscopy was applied to better characterize this process.

Mitophagic flux was measured by flow cytometry as the ratio of the mitochondrial probe MitoTracker Deep Red (MTDR; Molecular Probes) fluorescence in the presence of the lysosomal inhibitor chloroquine (CQ; Molecular Probes) to the MTDR in the absence of the inhibitor, normalized to the corresponding value in control cells. For all experimental conditions, cells were incubated with 50nM MTDR for 15 min at 37°C and then treated w/o 30μM CQ, 2h before flow cytometry analyses [25].

### Intracytoplasmic detections: LAMP-3/CD63 and LAMP-1/CD107a

We performed tests on the endo-lysosomal network by CD63 and CD107a staining, using anti-CD63 and anti-CD107a antibodies which are respectively endosome and lysosome markers [77]. Using flow cytometry, we can measure the intracellular content of these two molecules with previous cellular fixation and permeabilization (FIX & PERM^®^ cell fixation and cell permeabilization kit—Invitrogen) [78]. Niemann-Pick and wild-type B lymphocytes were washed in PBS, resuspended in 250μl of FIX reagent and incubated at 4°C for 30 min. Then the cells were washed in the perm/washing buffer and resuspended in 250μl of PERM reagent. Mouse monoclonal anti-Human Antibodies anti-CD63 (clone TEA3/18, Immunostep) and anti-CD107a (clone H4A3, BioLegend), FITC- and PerCP/Cy5.5-conjugated respectively, were added to the bottom of the tube and incubated at 4°C for 30 min, at concentrations indicated in the manufacturer’s instructions. After a washing step, the supernatant was discarded and samples were acquired by flow cytometry, collecting at least 10,000 events for each tube.

### Flow cytometric measurement of CD107a surface expression (lysosomal exocytosis assay)

Cell surface CD107a (LAMP-1), which is found on lysosomes and intracellular lytic granules that are derived from lysosomes, was measured. When these vacuoles fuse with the cell membrane, their surface becomes part of the cell surface [79,80]. Thus, surface expression of CD107a was used as a marker for granule fusion with the cell membrane and lysosomal exocytosis. CD107a-PerCP/Cy5.5 antibody (clone H4A3, BioLegend) was added directly to 50μl of cellular suspension at a concentration indicated in the manufacturer’s instructions. Cells were incubated for 1h at RT and then acquired by flow cytometry.

### Evaluation of intracellular lipid content

For evaluation of lipid content, samples were stained with the red fluorescence dye 4,4-difluoro-3,5-bis(4-phenyl-1,3-butadienyl)-4-bora-3a,4a-diaza-s-indacene (BODIPY^®^ 665/676; Molecular Probes) and the hydrophobic dye Nile Red (NR; Sigma-Aldrich).

#### BODIPY^®^ 665/676 staining

BODIPY dyes are commonly used as stains for neutral lipids and as tracers for oils and other non-polar liquids. BODIPY^®^ 665/676 (BP) specifically has a high affinity for free fatty acids and triglycerides, with a long-wave absorption maximum of 665 nm, and a fluorescence emission maximum of 676 nm. Furthermore, lipid peroxidation can be detected with this lipophilic probe that exhibits a change in fluorescence after interaction with peroxyl radicals [81]. For cytometric analysis of neutral lipid content and peroxidation, cells were loaded with BODIPY^®^ 665/676 at the final concentration of 1μg/ml for 30 minutes at 37°C. Following incubation, samples were acquired by FACSCalibur flow cytometer. In addition, to clearly understand the relationship between lipid/lysosomes and the lipid degradation pathways, we performed a co-labeling BP/LTG. Cells were stained with LTG 500nM for 45 minutes at 37°C. During the last 30 min of incubation, cells were exposed to BP 1μg/ml, re-cultured at 37°C for 30 minutes and then analyzed by a Leica TCS SP5 II confocal microscope. Colocalization analyses (Pearson's correlation coefficient) were performed using JACoP plugin in ImageJ [76].

#### Nile Red staining

Nile Red (NR) is a phenoxazine dye that can be used on living cells to localize and quantify neutral and polar lipids. Polar lipids (i.e., phospholipids) which are mostly present in membranes, are stained in red (emission > 590 nm), whereas neutral lipids (esterified cholesterol and triglycerides), which are present in lipid droplets, are stained in yellow (570–590 nm) [75,82,83]. In the present investigation, NR was prepared at 100μg/ml in dimethylsulfoxide. NR was added to the culture medium for 15 min at a final concentration of 1μg/ml in a cellular suspension adjusted to 10^6^ cells/ml. After incubation, samples were acquired by FC, collecting at least 10,000 events for each tube, and analyzed by confocal microscopy.

### Microvesicle/microparticle labeling: CD63 and lipid probes

In order to detect microparticles in the extracellular environment, without any preparation step of ultracentrifugation, we labelled each sample (100μl) by means of 20μl of anti-CD63 FITC-conjugated (Immunostep) mAb. After an incubation of 60 min to ensure the binding of the monoclonal antibody (mAb) to the specific epitope, which is generally borne by exosomes and other microvesicles, we proceeded with FC analyses. The flow cytometry approach consists of acquiring samples mixed with beads of defined size (Ø 0.5 μm, 1 μm, 5.2 μm) to obtain a size calibration of small particles detected outside the scatter area of intact cells. Furthermore, Nile Red (Sigma-Aldrich) was likewise added to samples, always taking into account both particles and cell fluorescence and absolute counting. It is important to specify that FCS of the complete medium was ultracentrifuged to minimize contamination by serum microvesicles. Samples were acquired by a FACSCanto flow cytometer and FACSDiva^™^ software was used to analyze all of the data from these flow cytometric experiments.

### Transmission Electron Microscopy (TEM)

Electron microscopy represents a reliable method to monitor autophagy stages and it is still indispensable for the characterization of autophagic organelles. To evaluate morphological aspects, pellets were washed and immediately fixed “in situ” with 2.5% glutaraldehyde in 0.1M phosphate buffer for 45 min, post-fixed for 1h in 1% OsO_4_, alcohol dehydrated, and embedded in araldite. Thin sectioning was preceded by the analysis of toluidine blue stained semithin sections by light microscopy. Thin sections were stained with uranyl acetate and lead citrate and analyzed with a Philips CM10 transmission electron microscope [84].

### Flow cytometric analyses

Cytometric experiments were carried out with a FACSCalibur flow cytometer (BD Biosciences) equipped with a blue laser (488 nm, air-cooled, 15 mW Argon) and a red diode laser (633 nm), a FACSCanto flow cytometer (BD Biosciences) equipped with blue (488 nm, air-cooled, 20 mW solid state) and red (640 nm, 40 mW solid state) lasers, and a FACSCanto II flow cytometer (BD Biosciences) equipped with blue (488 nm, air-cooled, 20 mW solid state), red (633 nm, 17 mW HeNe), and violet (405 nm, 30 mW solid state) lasers. Analyses were performed with CellQuest^™^ (BD Biosciences) and FACSDiva^™^ software (BD Biosciences). Samples were acquired by flow cytometry, collecting at least 10,000 events for each tube. Starved cells (partially adherent to flask), after staining with dyes described above, were rinsed twice with PBS and harvested by standard trypsinization (0.5 mg/ml trypsin and 0.2 mg/ml EDTA in PBS; Sigma-Aldrich). Detached cells were pooled with supernatants, pelleted by centrifugation, and washed with PBS. Cells were finally resuspended in PBS before flow cytometric analysis.

### Fluorescence and confocal microscopy

Fluorescence and light microscopy analyses were performed by a Nikon TS100 fluorescence microscope (Nikon Corporation) and a Leica TCS SP5 II confocal microscope (Leica Microsystem) with 488, 543 and 633 nm illumination and oil-immersed objectives. For confocal live imaging, cells were grown on MatTek glass bottom chambers (MatTek Corporation). The images were further processed and analyzed for mean fluorescence quantification in ImageJ software (National Institutes of Health), using the JACoP plug-in for colocalization studies [76]. We used the Pearson's coefficient as the parameter to measure colocalization in our samples.

### Statistical analysis

Data are shown as mean ± standard deviation (SD) of at least three independent experiments. Analyses of variance (ANOVA) approaches were used to compare values among different experimental groups for data that met the normality assumption. Differences between groups were analyzed by using a one-way analysis of variance (One-way ANOVA), followed by a Tukey *post hoc* analysis. Two-way ANOVA was used to compare cell lines and treatments as separate factors and then Bonferroni *post hoc* tests were used to compare cell lines for each treatment. *P* values less than 0.05 were considered statistically significant. All statistical analysis was done using GraphPad Prism 5.0 (GraphPad software).

## Supporting Information

S1 Fig Cytometric analyses of mitochondrial and lysosomal compartments. (**A**) Statistical histogram of MFI variation of MitoTracker Green (MTG) in tWT and tNP cells for each experimental condition. Each value is expressed as a mean ± SD (Results from n ≥ 3 independent experiments). The difference between cell lines was significant as shown by two-way ANOVA (***P < 0.001). (**B**) Contour plot LysoTracker Green (LTG) *vs* MitoTracker Red (MTR) from tNP cells for control, starved and nocodazole-treated cells.
(TIF)

S2 Fig Autophagic vacuoles, autophagosomes and endolysosomal compartment analyses. (**A**) Cytometric histograms relative to AO FL3 Mean Intensity Fluorescence (MFI) of all experimental conditions. (**B**) Statistical histogram of MFI expression LAMP-1/CD107a in tWT and tNP cells. Mean values were converted to arbitrary units (A.U.) setting control of wild-type cells as 100. Each value is expressed as a relative mean ± SD (Results from n ≥ 3 independent experiments). The difference between cell lines was significant as shown by two-way ANOVA (***P < 0.001). (**C**) Statistical histogram of MFI basal expression LAMP-3/CD63 in tWT and tNP cells. Mean values were converted to arbitrary units (A.U.) setting control of wild-type cells as 100. Each value is expressed as a relative mean ± SD (Results from n ≥ 3 independent experiments); *P < 0.05 *vs* tWT control. (**D-E**) Autophagosome detection by LC3B-GFP in confocal microscopy. (**D**) Single confocal optical sections (~0.8 μm thickness) showing LC3B-GFP positive puncta from control and rapamycin-treated tWT and tNP cells. Bars: 10 μm; 5 μm for insets. (**E**) Statistical histogram depicting MFI variation of LC3B-GFP in tWT and tNP cells for control and rapamycin conditions obtained from confocal microscopy images by ImageJ software. Mean values were converted to arbitrary units (A.U.) setting control of wild-type cells as 100. Each value is expressed as a relative mean ± SD (Results from n ≥ 3 independent experiments); *P < 0.05 *vs* tWT control.
(TIF)
