# Supplementary figures and images for: Defective Autophagy, Mitochondrial Clearance and Lipophagy in Niemann-Pick Type B Lymphocytes
Source: PLoS One. 2016 Oct 31;11(10):e0165780. doi: 10.1371/journal.pone.0165780 (PMC5087958; doi:10.1371/journal.pone.0165780)

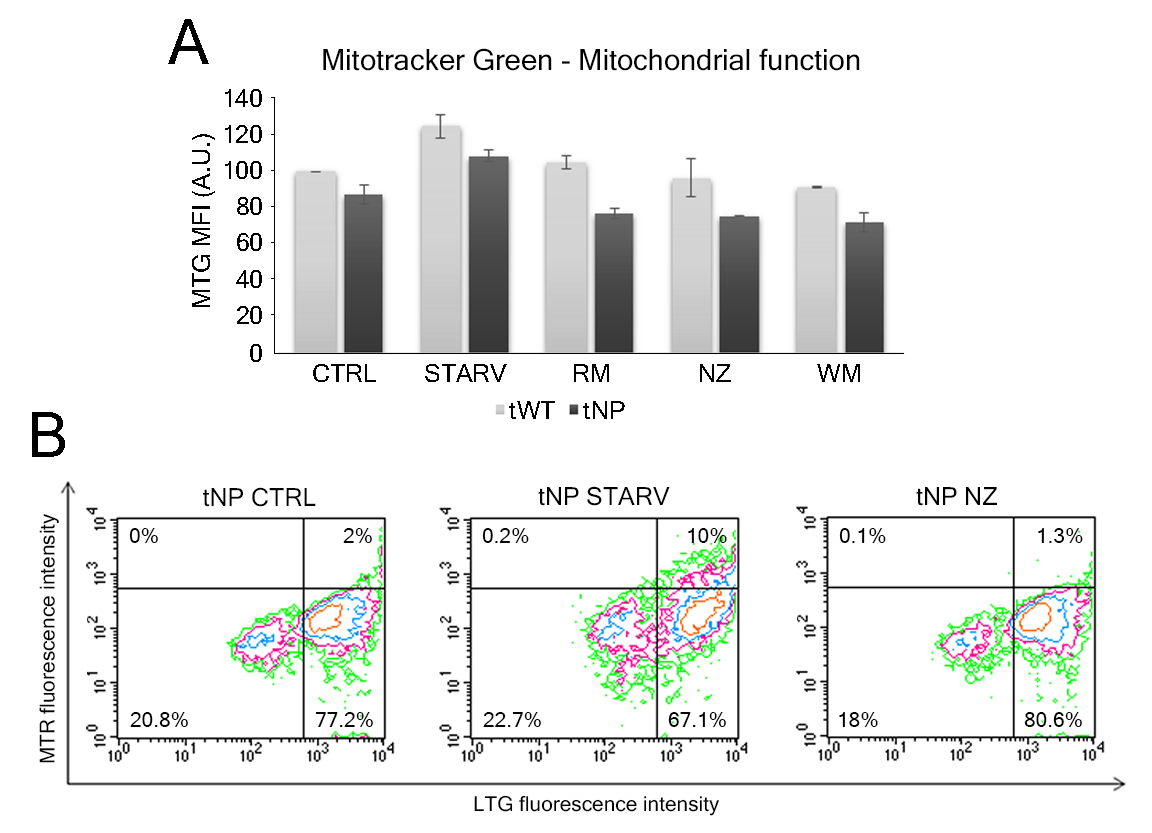

Supplement: S1 Fig — (A) Statistical histogram of MFI variation of MitoTracker Green (MTG) in tWT and tNP cells for each experimental condition. Each value is expressed as a mean ± SD (Results from n ≥ 3 independent experiments). The difference between cell lines was significant as shown by two-way ANOVA (***P < 0.001). (B) Contour plot LysoTracker Green (LTG) vs MitoTracker Red (MTR) from tNP cells for control, starved and nocodazole-treated cells. (TIF) [file pone.0165780.s001.tif]

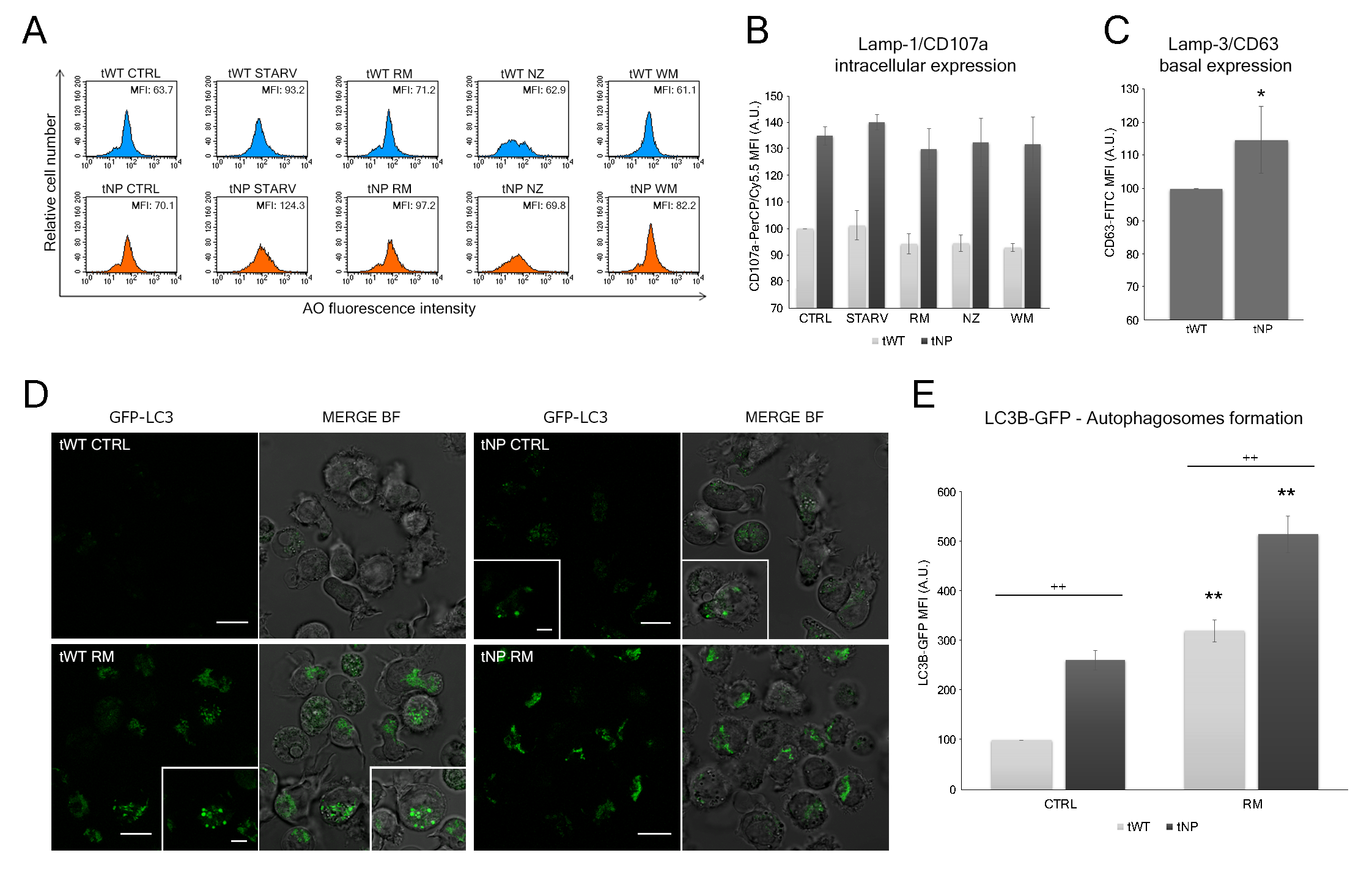

Supplement: S2 Fig — (A) Cytometric histograms relative to AO FL3 Mean Intensity Fluorescence (MFI) of all experimental conditions. (B) Statistical histogram of MFI expression LAMP-1/CD107a in tWT and tNP cells. Mean values were converted to arbitrary units (A.U.) setting control of wild-type cells as 100. Each value is expressed as a relative mean ± SD (Results from n ≥ 3 independent experiments). The difference between cell lines was significant as shown by two-way ANOVA (***P < 0.001). (C) Statistical histogram of MFI basal expression LAMP-3/CD63 in tWT and tNP cells. Mean values were converted to arbitrary units (A.U.) setting control of wild-type cells as 100. Each value is expressed as a relative mean ± SD (Results from n ≥ 3 independent experiments); *P < 0.05 vs tWT control. (D-E) Autophagosome detection by LC3B-GFP in confocal microscopy. (D) Single confocal optical sections (~0.8 μm thickness) showing LC3B-GFP positive puncta from control and rapamycin-treated tWT and tNP cells. Bars: 10 μm; 5 μm for insets. (E) Statistical histogram depicting MFI variation of LC3B-GFP in tWT and tNP cells for control and rapamycin conditions obtained from confocal microscopy images by ImageJ software. Mean values were converted to arbitrary units (A.U.) setting control of wild-type cells as 100. Each value is expressed as a relative mean ± SD (Results from n ≥ 3 independent experiments); *P < 0.05 vs tWT control. (TIF) [file pone.0165780.s002.tif]
